# Prediction of circRNA-disease associations based on inductive matrix completion

**DOI:** 10.1186/s12920-020-0679-0

**Published:** 2020-04-03

**Authors:** Menglu Li, Mengya Liu, Yannan Bin, Junfeng Xia

**Affiliations:** 1 Key Laboratory of Intelligent Computing and Signal Processing of Ministry of Education, School of Computer Science and Technology, Anhui University, Hefei, 230601 Anhui China; 2 Institutes of Physical Science and Information Technology, Anhui University, Hefei, 230601 Anhui China

**Keywords:** CircRNA-disease associations, CircRNA sequence similarity, Disease semantic similarity, Inductive matrix completion

## Abstract

**Background:**

Currently, numerous studies indicate that circular RNA (circRNA) is associated with various human complex diseases. While identifying disease-related circRNAs in vivo is time- and labor-consuming, a feasible and effective computational method to predict circRNA-disease associations is worthy of more studies.

**Results:**

Here, we present a new method called SIMCCDA (Speedup Inductive Matrix Completion for CircRNA-Disease Associations prediction) to predict circRNA-disease associations. Based on known circRNA-disease associations, circRNA sequence similarity, disease semantic similarity, and the computed Gaussian interaction profile kernel similarity, we used speedup inductive matrix completion to construct the model. The proposed SIMCCDA method obtains an area under ROC curve (AUC) of 0.8465 with leave-one-out cross validation in the dataset, which is obtained by the combination of the three databases (circRNA disease, circ2Disease and circR2Disease). Our method surpasses other state-of-art models in predicting circRNA-disease associations. Furthermore, we conducted case studies in breast cancer, stomach cancer and colorectal cancer for further performance evaluation.

**Conclusion:**

All the results show reliable prediction ability of SIMCCDA. We anticipate that SIMCCDA could be utilized to facilitate further developments in the field and follow-up investigations by biomedical researchers.

## Background

As endogenous noncoding RNA, circular RNA (circRNA) is extremely distinct from linear RNA. The largest difference is that the circRNA does not possess a terminal structure (i.e., 5′ caps and 3′ polyA tails) and is covalently closed to form a loop structure [1]. Such a loop structure facilitates the resistance of the circRNA to the degradation of RNA exonuclease and offers a stable biological effect compared with the corresponding linear structure [2, 3].

Although circRNA was discovered as early as the 1970s, it was considered ‘junk’ RNA [4]. Recently, circRNA has been re-recognized and has gradually gained attention. CircRNA is involved in numerous important biological functions, especially regulatory functions [5]. Accumulating evidence has clearly demonstrated that changes in circRNA plays an important role in developing various pathological conditions and exhibits a significant correlation with diseases, especially cancer. For example, the circRNA CDR1as is an inhibitor of miR-7, which is known to be involved in various diseases, such as neurodegenerative diseases, atherosclerosis and breast cancer [6]. Therefore, circRNA is thought to be a promising disease biomarker and treatment target [5]. Analysis of existing circRNA-disease associations is necessary to help predict other potential associations and help us understand the molecular mechanisms of human disease and identify biomarkers for disease diagnosis, treatment, and prevention at the circRNA level [7].

To date, an increasing number of experimentally verified or reported databases are available for the circRNA-disease associations, such as circR2Disease [7], circRNA disease [8], circ2Disease [9], and circ2Traits [10]. However, experimental methods are too expensive and time-consuming to obtain a large validated circRNA-disease association data. Developing computational methods to predict novel circRNA-disease associations has attracted considerable attention as they can effectively decrease the time and cost of biological experiments. In addition, few methods are available for predicting the circRNA-disease associations using computational methods. Lei et al. [11] developed the method of predicting circRNA-disease associations based on a path weighted model, and Fan et al. [12] proposed the KATZHCDA method using the KATZ model on heterogeneous networks. However, these methods predict potential associations using a single database, which is not enough to illustrate the stability of the model. Moreover, it remains challenging to achieve significant performance for predicting circRNA-disease associations.

In this work, we proposed a new method called SIMCCDA (Speedup Inductive Matrix Completion for CircRNA-Disease Associations prediction), which considers the prediction of circRNA-disease associations as a recommendation system problem. To the best our knowledge, we are the first to apply the recommendation system approach inductive matrix completion (IMC) [13–15] to predict circRNA-disease associations. This method has been applied for various bioinformatics problems, such as drug-target interactions [16], drug repositioning [17], lncRNA (long non coding RNA)-disease [18] and miRNA (microRNA)-disease associations [19]. We model the circRNA-disease association prediction problem as a recommendation task and solve it using speedup IMC [20]. Three databases, including circRNA disease, circ2Disease and circR2Disease, are used as our raw data in this study. We then perform data screening, generate corresponding three sub-datasets (Dataset-1, Dataset-2 and Dataset-3), and combine them into a total dataset (named TotalCircRD-1). We first calculate circRNA sequence similarity and disease semantic similarity in these four datasets. Next, these two types of similarities are combined into a Gaussian interaction profile kernel to generate new circRNA similarity and disease similarity. Primary feature vectors of the similarity matrix are extracted by principal component analysis (PCA). The final model based on IMC is built for predicting circRNA-disease associations.

Leave-one-out cross validation (LOOCV) is used to examine the performance of our method. The optimal AUC on TotalCircRD-1 is 0.8465. The AUC results on the three datasets are 0.8682 (Dataset-1), 0.8303 (Dataset-2) and 0.8509 (Dataset-3), respectively. To further evaluate the performance of the proposed method, we rank and select the top 30 predictions of each dataset to determine the number of results that existed in verified associations. We also conduct case studies in breast cancer, stomach cancer and colorectal cancer to support our predictions. Finally, we compare our method with KATZHCDA, and the prediction results indicate that our method outperforms the previous method in predicting circRNA-disease associations. In summary, the proposed SIMCCDA method has the ability to predict associations in circRNA-disease and offers a guiding significance for future biomedical clinical experiments.

## Methods

### Model overview

**Fig. 1:**
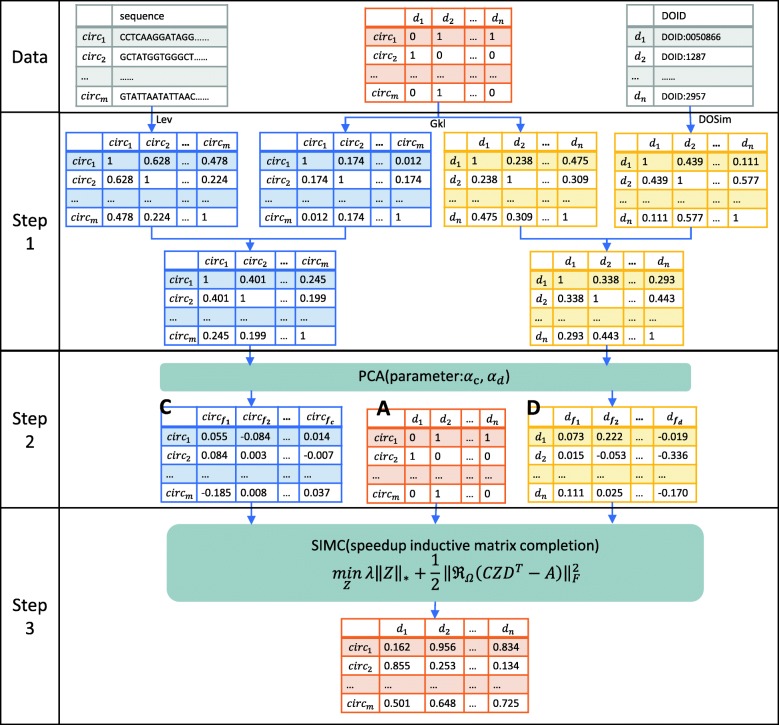
The overall procedure of SIMCCDA. Step 1: compute circRNA similarity and disease similarity. Step 2: extract primary feature vectors. Step 3: predict the circRNA-disease association matrix with speedup IMC. Lev: Levenshtein distance, Gkl: Gaussian interaction profile kernel

Here, we apply IMC with feature vectors to build the model called SIMCCDA. In addition, we add a linear Bregman iteration to speed up the process of calculating the final score matrix. The flowchart is presented in Fig. 1. *A*_*ij*_ = 1 indicates that circRNA *circ*_*i*_ and disease *d*_*j*_ are associated, whereas *A*_*ij*_ = 0 indicates that their association is currently in an unknown state. Given a known circRNA-disease association matrix *A* ∈ *ℝ*^*m* × *n*^ with circRNA sequences and disease DOIDs (disease ontology identities), we obtain circRNA and disease similarity, respectively. Then, PCA is employed to extract primary feature vectors from acquired similarity. Finally, we construct the model with IMC based on the above information to predict circRNA-disease associations.

### Human circRNA-disease associations data

We use three databases, including circRNA disease, circ2Disease and circR2Disease, all of which include known human circRNA-disease associations. All data were downloaded before September 2018. The initial information regarding each downloaded dataset is as follows: the first database circRNA disease contains 354 circRNA-disease associations (including 330 circRNAs and 48 diseases), the second database circ2Disease includes 273 circRNA-disease associations (including 249 circRNAs and 61 diseases) and the third database circR2Disease includes 739 associations (including 661 circRNAs and 100 diseases). The sequence information of circRNA and disease DOID matching are applied to the circBase [21] and Disease Ontology [22] (DO) databases. Based on the above data processing, we generate the final three datasets (Dataset-1, Dataset-2 and Dataset-3). These datasets are merged to obtain TotalCircRD-1 without duplicated redundancy. Table 1 lists the detailed statistics of the four datasets.

**Table 1:** Details of four datasets

Dataset	Number of circRNAs	Number of diseases	Number of associations	Matrix density
Dataset-1	223	34	241	0.032
Dataset-2	215	46	240	0.024
Dataset-3	389	61	445	0.019
TotalCircRD-1	512	71	609	0.017

The uncompleted associations in the datasets include circRNAs without sequences or diseases without DOIDs. Given that the calculation of circRNA sequence similarity requires the circRNA sequence and the disease similarity requires the disease DOID information, the preceding datasets exclude the uncompleted associations. We wanted to assess whether these uncompleted associations would influence the prediction performance, so we add several uncompleted associations to form four new datasets (Dataset-4, Dataset-5, Dataset-6 and TotalCircRD-2) based on Dataset-1, Dataset-2, Dataset-3 and TotalCircRD-1, respectively (Additional file 1: Table S1).

### CircRNA sequence similarity

The sequence information of all the corresponding circRNAs in the aforementioned databases is obtained from circBase, and Levenshtein distance [23] is used to calculate the similarity between each two circRNA sequences. As a string metric for measuring the difference between two strings, the Levenshtein distance between two strings is the minimum cost of single-character edits (insertions, deletions or replacements) required to change one string into the other. In the present study, both editing costs of insertion and deletion are 1, and the replacement editing cost is 2. Formula (1) is the calculation of similarity for two circRNA sequences:

1 $$ Si{m}_{lev}\left( cir{c}_i, cir{c}_j\right)=1-\frac{dist}{len\left( cir{c}_i\right)+ len\left( cir{c}_j\right)} $$

where *dist* represents the minimum editing cost of converting the circRNA *circ*_*i*_ sequence to the circRNA *circ*_*j*_ sequence, and *len*(∙) represents the length of circRNA sequence.

### Disease semantic similarity

We use DOSim [24] in DO-based DOSE (R package) to calculate the disease semantic similarity with Wang measure [25]. The detailed formula is displayed as follow:

2 $$ {Sim}_{Wang}\left({d}_i,{d}_j\right)=\frac{\sum_{t\in {T}_{d_i}\cap {T}_{d_j}}\left({S}_{d_i}(t)+{S}_{d_j}(t)\right)}{\sum_{t\in {T}_{d_i}}{S}_{d_i}(t)+{\sum}_{t\in {T}_{d_j}}{S}_{d_j}(t)} $$

For a given disease *d*_*i*_, ${T}_{d_i}$ is the ancestor term set of term *d*_*i*_ (including *d*_*i*_ itself). ${S}_{d_i}(t)$ is defined as the contribution score of disease *t* ($t\in {T}_{d_i}$) to disease *d*_*i*_. It can be expressed by the following formula:

3 $$ \Big\{{}_{S_{d_i}(t)=\max \left\{{w}_e\times {S}_{d_i}\left({t}^{\prime}\right)|{t}^{\prime}\in childrenof(t)\right\}\  if\ t\ne {d}_i}^{S_{d_i}\left({d}_i\right)=1} $$

Here, *w*_*e*_ is the semantic contribution factor of edge *e*, where *e* belongs to the set of edges connecting *d*_*i*_ and its ancestor ${T}_{d_i}$. In DOSim, we set *w*_*e*_ = 0.7.

### Gaussian interaction profile kernel similarity for circRNA and disease

By considering the assumption that similar circRNAs tend to be bound with similar diseases, Gaussian interaction profile kernel similarity is computed based on the known circRNA-disease association datasets. Inspired by van Laarhoven et al. [26], we calculate the circRNA and disease similarity using the Gaussian interaction profile kernel on four datasets. Equations (4) and (5) determine the similarity between *circ*_*i*_ and *circ*_*j*_, where *m* is circRNA number, *IP*(*circ*_*i*_) is the associated disease set corresponding to the *circ*_*i*_, and *γ*_*c*_ is the regulation parameter of kernel bandwidth.

4 $$ Gkl\left({circ}_i,{circ}_j\right)=\exp \left(-{\gamma}_c{\left\Vert I\mathrm{P}\left({circ}_i\right)- IP\left({circ}_j\right)\right\Vert}^2\right) $$

5 $$ {\gamma}_c=\frac{1}{\frac{1}{m}{\sum}_{i=1}^m{\left\Vert IP\left({circ}_i\right)\right\Vert}^2} $$

The Gaussian interaction profile kernel similarity of diseases *d*_*i*_ and *d*_*j*_ is similar to the defined equations (6) and (7), where *n* is the number of diseases:

6 $$ Gkl\left({d}_i,{d}_j\right)=\exp \left(-{\gamma}_d{\left\Vert IP\left({d}_i\right)- IP\left({d}_j\right)\right\Vert}^2\right) $$

7 $$ {\gamma}_d=\frac{1}{\frac{1}{n}{\sum}_{i=1}^n{\left\Vert IP\left({d}_i\right)\right\Vert}^2} $$

### Integrated similarity for circRNA and disease

Based on the previously defined circRNA sequence similarity, disease semantic similarity and Gaussian interaction profile kernel similarities, the integrated circRNA similarity matrix *CS* and the disease similarity matrix *DS* are calculated using the following equations (8) and (9):

8 $$ CS\left({circ}_i,{circ}_j\right)=\frac{Sim_{lev}\left({circ}_i,{circ}_j\right)+ Gkl\left({circ}_i,{circ}_j\right)}{2} $$

9 $$ DS\left({d}_i,{d}_j\right)=\frac{Sim_{Wang}\left({d}_i,{d}_j\right)+G\mathrm{k}l\left({d}_i,{d}_j\right)}{2} $$

### Extract primary feature vectors

To remove the similarity redundancy, we use principal component analysis (PCA) to extract the primary feature vectors from integrated similarity, *CS* and *DS*. In this method, based on the dominating energy strategy [27], we use singular value decomposition (SVD) to perform PCA and formulas (10) and (11) to obtain the primary feature vectors of circRNA and disease similarity.

10 $$ \underset{f_c}{\arg\ \min}\left\{\frac{\sum_{i=1}^{f_c}{\left({S}_c\right)}_{ii}}{\sum_{j=1}^m{\left({S}_c\right)}_{jj}}\ge {\alpha}_c\right\} $$

11 $$ \underset{f_d}{\arg\ \min}\left\{\frac{\sum_{i=1}^{f_d}{\left({S}_d\right)}_{ii}}{\sum_{j=1}^n{\left({S}_d\right)}_{jj}}\ge {\alpha}_d\right\} $$

In the above formulas, *S*_*c*_ and *S*_*d*_ are the singular values of circRNA and the disease similarity matrix, respectively. *α*_*c*_ and *α*_*d*_ are adjusted parameters to obtain optimal results. In this study, Dataset-2, Dataset-3 and TotalCircRD-1 share the parameters *α*_*c*_ =0.6 and *α*_*d*_ =0.9, whereas the parameters of Dataset-1 are *α*_*c*_ =0.7 and *α*_*d*_ =0.9. Detailed adjustment work of *α*_*c*_ and *α*_*d*_ is discussed in the Results section.

### Model construction

In this study, we formulate circRNA-disease association prediction as a recommendation system problem. Generally, a recommendation system is an information filtering system that seeks to predict the user’s preference of a certain item based on partial known preference information. We here use the recommendation system method IMC [15] to identify circRNAs for a disease that is dependent on validated circRNA-disease associations.

Observing the matrix density of the last column in Table 1, we find that the association matrix is very sparse. As we know, there are a small amount of experimental data of associations due to the structural complexity of circRNAs and ignored biological functions. The available data scale is in the primary stage. As a result, we cover the unknown associations of circRNAs and diseases through IMC to enhance the quality of our data. The advantage is that IMC can solve matrix completion problems using a relatively small set of known information. The detailed process of IMC is described below. First, based on the assumption that the human circRNA-disease association matrix is *A*, the row vectors in *A* lie in the subspace spanned by the column vectors in *D* (disease feature vectors), and the column vectors in *A* lie in the subspace spanned by the column vectors in *C* (circRNA feature vectors). The problem can be defined as:

12 $$ \underset{Z\in {\Re}^{f_c\times {f}_d}}{\min}\lambda {\left\Vert Z\right\Vert}_{\ast }+\frac{1}{2}{\left\Vert {\Re}_{\varOmega}\left( CZ{D}^T-A\right)\right\Vert}_F^2 $$

where *Z* is the objective matrix to complete *A*, *CZD*^*T*^ is the final scoring matrix based on the association matrix and the similarity matrix, *Ω* represents known association sets, ‖∙‖_∗_ is the nuclear norm defined as the sum of the singular values, λ is the regularization parameter controlling the extent of the nuclear norm (here we set λ to 1), and ‖∙‖_*F*_ is the Frobenius norm of the matrix.

Representing *f*(*Z*) as $\frac{1}{2}{\left\Vert {\mathfrak{R}}_{\varOmega}\left( CZ{D}^T-A\right)\right\Vert}_F^2$, the formula (12) can be expressed as:

13 $$ \underset{Z\in {\Re}^{f_c\times {f}_d}}{\min}\lambda {\left\Vert Z\right\Vert}_{\ast }+f(Z) $$

For any given $Y\in {R}^{f_c\times {f}_d}$, the following quadratic approximation of *f*(*Z*) at *Y* can be considered as:

14 $$ f(Z)\approx {f}_{\tau}\left(Z,Y\right)=f(Y)+\left\langle \nabla f(Y),Z-Y\right\rangle +\frac{\tau }{2}{\left\Vert Z-Y\right\Vert}_F^2+P(Z)=\frac{\tau }{2}{\left\Vert Z-\left(Y-\frac{1}{\tau}\nabla f(Y)\right)\right\Vert}_F^2+f(Y)-\frac{1}{2\tau}\left\Vert \nabla f{(Y)}_F^2\right\Vert $$

where $\nabla f(Y)={C}^T{\mathfrak{R}}_{\Omega}\left( CY{D}^T-A\right)D$ is the gradient of *f*(*Z*) at *Y*, 〈∙〉 represents matrix inner product, and *τ* is a proximal parameter for estimating the second-order gradient ∇^2^*f*(*Y*). Accordingly, the above formula (13) calculates the minimum model, which can be converted into the following formula:

15 $$ \underset{Z\in {\Re}^{f_c\times {f}_d}}{\min}\lambda {\left\Vert Z\right\Vert}_{\ast }+\frac{\tau }{2}{\left\Vert Z-\left(Y-\frac{1}{\tau}\nabla f(Y)\right)\right\Vert}_F^2 $$

Then, we use the accelerated proximal gradient singular value thresholding algorithm [28] with iterate *h* times to obtain *Z* [29].

**Figure:**
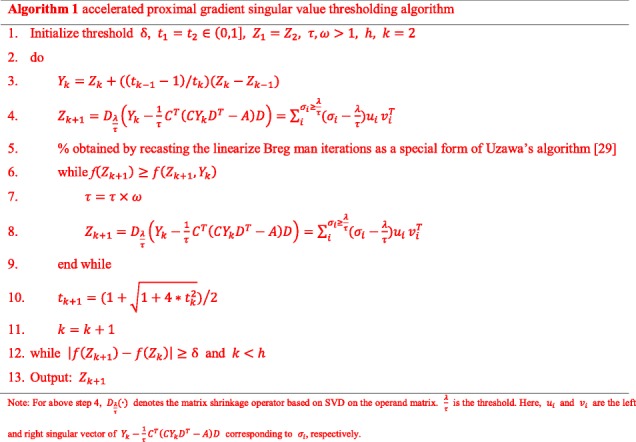

In order to see the relationship between the objective function value and the number of iterations, we divide the circRNAs into several categories according to their chromosomal location and then select randomly one from each class to view the trend of the curve. Additional file 1: Figure S1 shows that the value of the objective function decreases as the number of iterations increases. When the gap of objective function values between two iterations is particularly small, i.e. $1-\frac{{objective\ value}_k}{{objective\ value}_{k-1}}<{10}^{-5}$, the iterative process will end.

## Results

### LOOCV

To assess the predictive accuracy of SIMCCDA, we performed the following method using the leave-one-out cross validation (LOOCV) framework on the known circRNA-disease associations. The reason why LOOCV is used in this study is that the current common practice in this field (prediction of lncRNA/miRNA/circRNA-disease associations) [30–32] is to use LOOCV to measure the performance of the model. For a disease *d*_*i*_, each known circRNA association corresponding to the disease was left as a test sample. Other known associations were used as training samples, and an initial non-association was regarded as a candidate sample. In the candidate samples and test sample set, the test sample was deemed as a positive sample, and the others were negative samples. After running the model, the probabilities of associations between candidate samples and disease *d*_*i*_ were calculated. We took the highest values as the final score of the candidate sample among probabilities. Finally, we calculated the sensitivity and specificity as follows:

16 $$ sensitivity=\frac{TP}{TP+ FN} $$

17 $$ specificity=\frac{TN}{TN+ FP} $$

where TP indicate true positives, FP is false positives, TN refer to true negatives, and FN represent false negatives.

A Receiver Operating Characteristics (ROC) curve is drawn based on the LOOCV result. The X-axis of the ROC graph is the 1-specificity, and the Y-axis is the sensitivity. From the ROC curve, the Area Under ROC Curve (AUC) can be calculated as an evaluation measure for the model.

### The effect of adjusting parameters on the prediction result

**Fig. 2:**
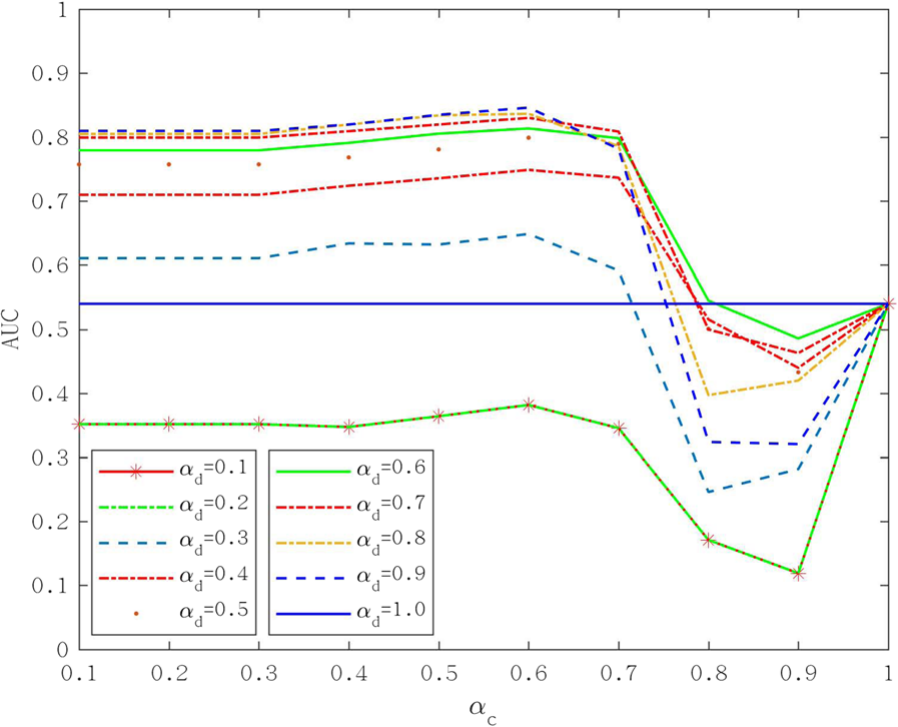
Adjusted parameters *α*_*c*_ and *α*_*d*_ with their impact on TotalCircRD-1 dataset

**Fig. 3:**
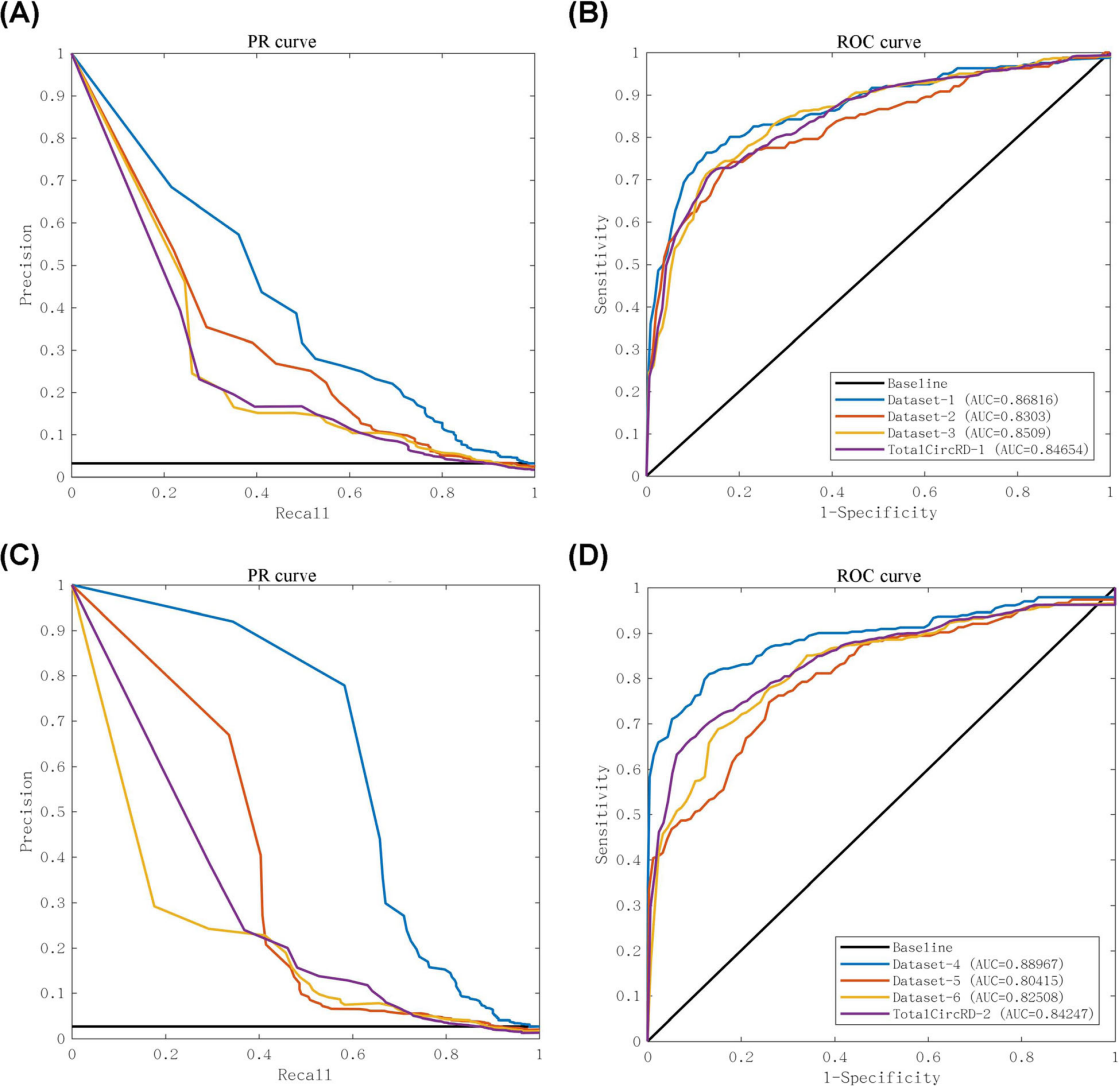
ROC curve and PR curve using LOOCV of eight datasets under optimal parameters. **a** PR curve in four datasets (Dataset-1, Dataset-2, Dataset-3 and TotalCircRD-1). **b** ROC curve in four datasets (Dataset-1, Dataset-2, Dataset-3 and TotalCircRD-1). **c** PR curve in four datasets (Dataset-4, Dataset-5, Dataset-6 and TotalCircRD-2). **d** ROC curve in four datasets (Dataset-4, Dataset-5, Dataset-6 and TotalCircRD-2)

**Fig. 4:**
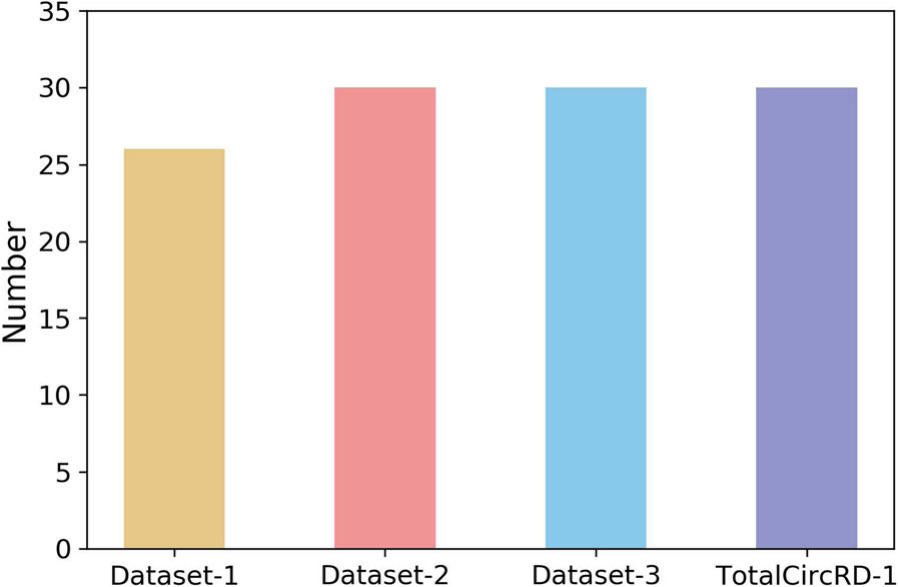
The number of associations validated by our model for the top 30 on four datasets (Dataset-1, Dataset-2, Dataset-3, and TotalCircRD-1)

In the PCA section of the Methods, two parameters *α*_*c*_ and *α*_*d*_ were included, which represent the percentage of singular values of circRNA and disease similarity matrix, respectively. We tried to take values between 0.1 and 1 for *α*_*c*_ and *α*_*d*_, and the step size was 0.1. The results of the parameterization of TotalCircRD-1 are presented in Fig. 2, and results for Dataset-1, Dataset-2 and Dataset-3 are presented in Additional file 1: Figures S2-S4. As noted in Fig. 2, as *α*_*c*_ increases, AUC is initially stable and the generally declines. The results are consistent when *α*_*d*_ =0.1 or *α*_*d*_ =0.2. As *α*_*d*_ increases, the AUC gradually increases, but the growth rate is slow. The optimal parameters of the three datasets of Dataset-2, Dataset-3, TotalCircRD-1 are all *α*_*c*_ =0.6 and *α*_*d*_ =0.9, whereas the optimal parameters for Dataset-1 are *α*_*c*_ =0.7 and *α*_*d*_ =0.9. LOOCV-based AUC results for four datasets with optimal parameters are shown in Fig. 3b. The results of our model on the four datasets are at a solid level, and the gap between the maximum and minimum values is 3% in four datasets, which reveals that our model exhibits better robustness. Figure 3a shows the PR (Precision-Recall) curves on the four datasets, respectively, which have the same trend as the ROC curve. Figure 4 presents the number of experimental validated associations predicted by our model from the top 30 predicted associations from our four datasets. Additional file 1: Table S2 shows the predicted results of the top 10, 30, 50 and 100. It can be observed that whether it is top 10, top 30, top 50, or top 100, the ultimate trends are similar. For the sake of convenience, we only show the results of top 30 in this work. Based on the above optimal parameters, we predicted 30 known circRNA-disease associations from Dataset-2, Dataset-3 and TotalCircRD-1, and 26 known associations from the Dataset-1. This shows that our results are optimal under these parameters, and four unknown associations in Dataset-1 may be potential associations based on subsequent analysis.

In addition, we added weights to each part of the integration similarity to see how the performance could be impacted. We added weights (range from 0 to 1) to *Sim*_*lev*_(*circ*_*i*_, *circ*_*j*_) and *Gkl*(*circ*_*i*_, *circ*_*j*_) in equation (8) and (9), respectively. For different weights circRNAs and diseases similarity, the final results were obtained by combining the two pairs. The Additional file 1: Figure S5 shows that the combinations of different similarity weights have similar results for the models obtained on different datasets. So, in the end, our model used equation (8) and (9) to respectively calculate the circRNAs similarity and diseases similarity.

### The effect of uncompleted associations

The *α*_*c*_ and *α*_*d*_ were adjusted in the same manner as described above, and the optimal parameters were selected to calculate the AUC in Dataset-4, Dataset-5, Dataset-6 and TotalCircRD-2 datasets, as presented in Fig. 3c and d. The AUC scores of new-added datasets (Fig. 3d) are slightly reduced compared with the initial datasets (Fig. 3b). Given that most of the newly added circRNA only involved in one disease, thus making the final association matrix sparser than previous one. For example, circ-BANP is only associated with colorectal cancer and is not associated with other diseases. Increasing association data are noted between circ-BANP and colorectal cancer, and the unknown associations of circ-BANP with other diseases also increase, as observed from the matrix density columns of Additional file 1: Table S1. In summary, uncompleted associations exhibit a minimal effect on the results and only slightly reduce the performance of predictions.

The above results show that the sparseness of the data set has little effect on the prediction results. But if the correlation matrix is too sparse, it will still affect the final prediction results. So our method has a premise that the association matrix cannot be too sparse. We conducted the following experiment to explore how the varying sparsity of datasets affect the overall performance. Since the final result has a certain relationship with the dataset, we performed sparsity processing on each dataset (0.002 was the step size, and the sparsity was reduced by 0.002 each time), respectively. The Additional file 1: Figure S6 shows that the result is not much changed when the sparsity of Dataset-1 is 0.015. But when the sparsity is 0.013, the performance starts to drop significantly. Similarly, for the other three datasets (Dataset-2, Dataset-3, TotalCircRD-1), the performance starts to drop significantly when the sparsity is 0.013, 0.009, and 0.007, respectively.

### Compared with the other method

**Fig. 5:**
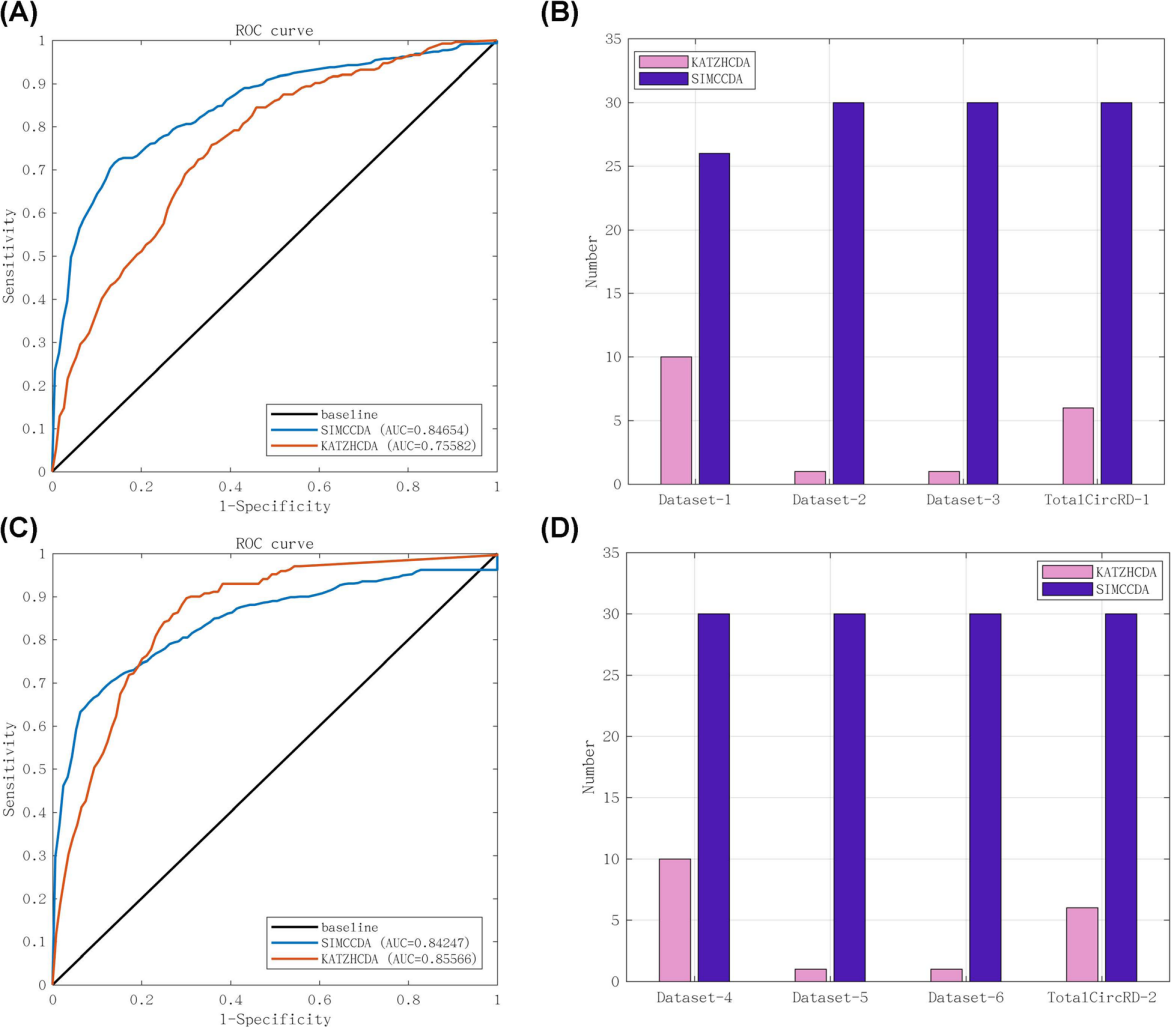
Comparison of SIMCCDA with KATZHCDA on the TotalCircRD-1 and Total CircRD-2 dataset. **a** Performance of methods in terms of ROC curve using LOOCV in TotalCircRD-1 dataset. **b** The number of experimental validations of the top 30 predicted circRNA-disease associations from four datasets (Dataset-1, Dataset-2, Dataset-3 and TotalCircRD-1). **c** Performance of methods in terms of ROC curve using LOOCV in TotalCircRD-2 dataset. **d** The number of experimental validations of the top 30 predicted circRNA-disease associations from four datasets (Dataset-4, Dataset-5, Dataset-6 and TotalCircRD-2)

Two methods are currently available for predicting circRNA-disease associations: PWCDA [11] and KATZHCDA [12]. Given that the PWCDA method needs to set the circRNA similarity and disease similarity < 0.5 part to 0 and most of the similarities on our datasets are less than 0.5, we only compared our method with KATZHCDA. KATZHCDA is a computational model of KATZ measures and constructs heterogeneous networks by employing the circRNA expression profiles, disease phenotype similarity and Gaussian interaction profile kernel similarity. Here, we used the same eight datasets in KATZHCDA model and obtain predicted results. The results of six datasets (Dataset-1, Dataset-2, Dataset-3, Dataset-4, Dataset-5 and Dataset-6) are presented in Additional file 1: Figure S7, and TotalCircRD-1 and TotalCircRD-2 results are presented in Fig. 5a and c. As shown in Additional file 1: Figure S7, both the PR curve and the ROC curve indicate that our model performance is superior to KATZHCDA. The AUC scores of four datasets (Dataset-1, Dataset-2, Dataset-3 and TotalCircRD-1) are 0.7604, 0.7458, 0.7442 and 0.7558, respectively. According to the comparison of two methods, our model obtains an average AUC of 0.8490, which is 9% higher than KATZHCDA. The resulting top 30 predicted associations are also analyzed, demonstrating that our predicted top 30 results are superior to KATZHCDA (Fig. 5b, d).

In addition, we compared our method with KATZHCDA by using Dataset-1 as the training set and Dataset-2, Dataset-3 as the test set. As can be seen from Additional file 1: Figure S8, our performance is slightly better than KATZHCDA. Specifically, the early stage of KATZHCDA prediction effect is better than ours, but its accuracy is reduced in the prediction of later stages. A comprehensive look at the above two results, our model is superior to KATZHCDA on the whole.

## Case study

### Analysis of predicted circRNA-disease associations with experimental evidence from the TotalCircRD-1 dataset

To further measure the performance of SIMCCDA, case studies of three diseases, including breast cancer, stomach cancer and colorectal cancer, from the TotalCircRD-1 dataset were analyzed in detail. The top 30 predicted disease-related circRNAs by SIMCCDA and supporting evidence from PubMed are presented in Tables 2, 3 and 4.

**Table 2:** Top 30 candidate circRNAs for breast cancer

Rank	circRNA	Evidence (PMID)	Rank	circRNA	Evidence (PMID)
1	hsa_circ_0001875	28484086	16	hsa_circ_0000911	28744405
2	hsa_circ_0006054	28484086	17	hsa_circ_0092276	28803498
3	hsa_circ_0000098	28744405	18	hsa_circ_0008945	28744405
4	hsa_circ_0107327	29221160	19	hsa_circ_0003838	28803498
5	hsa_circ_0001785	29045858	20	hsa_circ_0004619	28484086
6	hsa_circ_0103038	29221160	21	hsa_circ_0033144	29221160
7	hsa_circ_0002874	28803498	22	hsa_circ_0001283	28744405
8	hsa_circ_0002220	29221160	23	hsa_circ_0057129	29221160
9	hsa_circ_0006528	28803498	24	hsa_circ_0001824	28484086
10	hsa_circ_0008717	28744405	25	hsa_circ_0085495	28803498
11	hsa_circ_0000893	28744405	26	hsa_circ_0000732	28744405
12	hsa_circ_0068033	29045858	27	hsa_circ_0086241	28803498
13	hsa_circ_0011946	29593432	28	hsa_circ_0003221	unconfirmed^a^
14	hsa_circ_0001982	28933584	29	hsa_circ_0018293	28744405
15	hsa_circ_0001667	28803498	30	hsa_circ_0093859	29593432

^a^without the evidence reported in literatures

**Table 3:** Top 30 candidate circRNAs for stomach cancer

Rank	circRNA	Evidence (PMID)	Rank	circRNA	Evidence (PMID)
1	hsa_circ_0084606	28544609	16	hsa_circ_0076304	28831102
2	hsa_circ_0000140	25689795	17	hsa_circ_0057104	28831102
3	hsa_circ_0008383	28761361, 28206972	18	hsa_circ_0138960	28980874
4	hsa_circ_0074362	28544609, 29240459	19	hsa_circ_0013048	28657541, 28206972
5	hsa_circ_0003159	28618205	20	hsa_circ_0003789	28544609
6	hsa_circ_0006022	28639908	21	hsa_circ_0035445	28544609
7	has_circ_0031027	28206972	22	hsa_circ_0058766	28831102
8	hsa_circ_0050547	28544609	23	hsa_circ_0001895	28443463
9	hsa_circ_0001546	28544609	24	hsa_circ_0005927	28737829
10	hsa_circ_0063809	28544609	25	hsa_circ_0076305	28831102
11	hsa_circ_0084720	28831102	26	hsa_circ_0006633	28656881
12	hsa_circ_0032821	28737829	27	hsa_circ_0000154	28544609
13	hsa_circ_0001539	28184940	28	hsa_circ_0006470	28544609
14	hsa_circ_0003707	28639908	29	hsa_circ_0001017	29098316
15	hsa_circ_0006127	28974900	30	hsa_circ_0003222	28893265

**Table 4:** Top 30 candidate circRNAs for colorectal cancer

Rank	circRNA	Evidence (PMID)	Rank	circRNA	Evidence (PMID)
1	hsa_circ_0000523	25624062	16	hsa_circ_0006174	28656150
2	hsa_circ_0000504	28656150	17	hsa_circ_0001724	29207676
3	hsa_circ_0002138	25624062	18	hsa_circ_0001451	26884878
4	hsa_circ_0000567	29333615	19	hsa_circ_0074806	28656150
5	hsa_circ_0007006	28656150	20	hsa_circ_0003221	unconfirmed^a^
6	hsa_circ_0024169	25624062	21	hsa_circ_0001577	unconfirmed^a^
7	hsa_circ_0082333	26138677	22	hsa_circ_0008509	28656150
8	hsa_circ_0087862	28656150	23	hsa_circ_0022080	28656150
9	hsa_circ_0005949	28656150	24	hsa_circ_0002024	unconfirmed^a^
10	hsa_circ_0007031	28656150	25	hsa_circ_0000172	unconfirmed^a^
11	hsa_circ_0008494	28656150	26	hsa_circ_0000677	27058418
12	hsa_circ_0000069	28003761	27	hsa_circ_0002768	unconfirmed^a^
13	hsa_circ_0048232	28656150	28	hsa_circ_0091017	unconfirmed^a^
14	hsa_circ_0003098	28103507	29	hsa_circ_0002702	27058418
15	hsa_circ_0074930	28656150	30	hsa_circ_0128454	unconfirmed^a^

^a^without the evidence reported in literatures

Breast cancer is the most common cancer and remains the leading cause of cancer death among women worldwide [33]. Among top 30 predicted candidate circRNA for breast cancer, 29 are associated with breast cancer in related studies (Table 2). For instance, hsa_circ_0001875 (top 1) is upregulated in breast cancer tissues compared with the normal breast tissue [34]. In addition, circRNA hsa_circ_0006054 (top 2) expression is significantly downregulated in breast cancer tissues compared with non-breast cancer tissues [34].

Gastric cancer is the second disease to lead cancer-related mortality and the fourth most frequent cancer globally [35]. Using the SIMCCDA method, we successfully predicted 30 of top 30 candidate circRNAs for gastric cancer (Table 3). Among them, CircRNA hsa_circ_0084606 (top 1) is one of the top 10 upregulated circRNAs in stomach cancer tissues [36], whereas hsa_circ_0000140 (top 2), a typical circular RNA, is significantly increased in stomach cancer tissues compared with paired adjacent non-tumorous tissues [37].

Colorectal cancer is the third most common cancer worldwide with 1.36 million people diagnosed in 2012 [38]. The inferred results cover 23 experimental verified associations out of the top 30 ranked predictions (Table 4). The evidence in the literature reveals that circRNA hsa_circ_0000523 exhibits significantly reduced expression in cancer compared with normal colorectal tissues. In colorectal cancer cells, the well-validated circRNA hsa_circ_0000504 is upregulated [39].

### Analysis of predicted circRNA-disease associations without experimental evidence from four datasets

Given that the top 30 well-validated associations were successfully investigated by our method using Dataset-2, Dataset-3 and TotalCircRD-1 dataset, here we concentrated on four new predicted potential circRNA-disease associations from Dataset-1 (as shown in Fig. 4). We employed circRNA-miRNA and miRNA-disease associations to construct corresponding circRNA-miRNA-mRNA networks for the four new circRNA-disease associations.

**Fig. 6:**
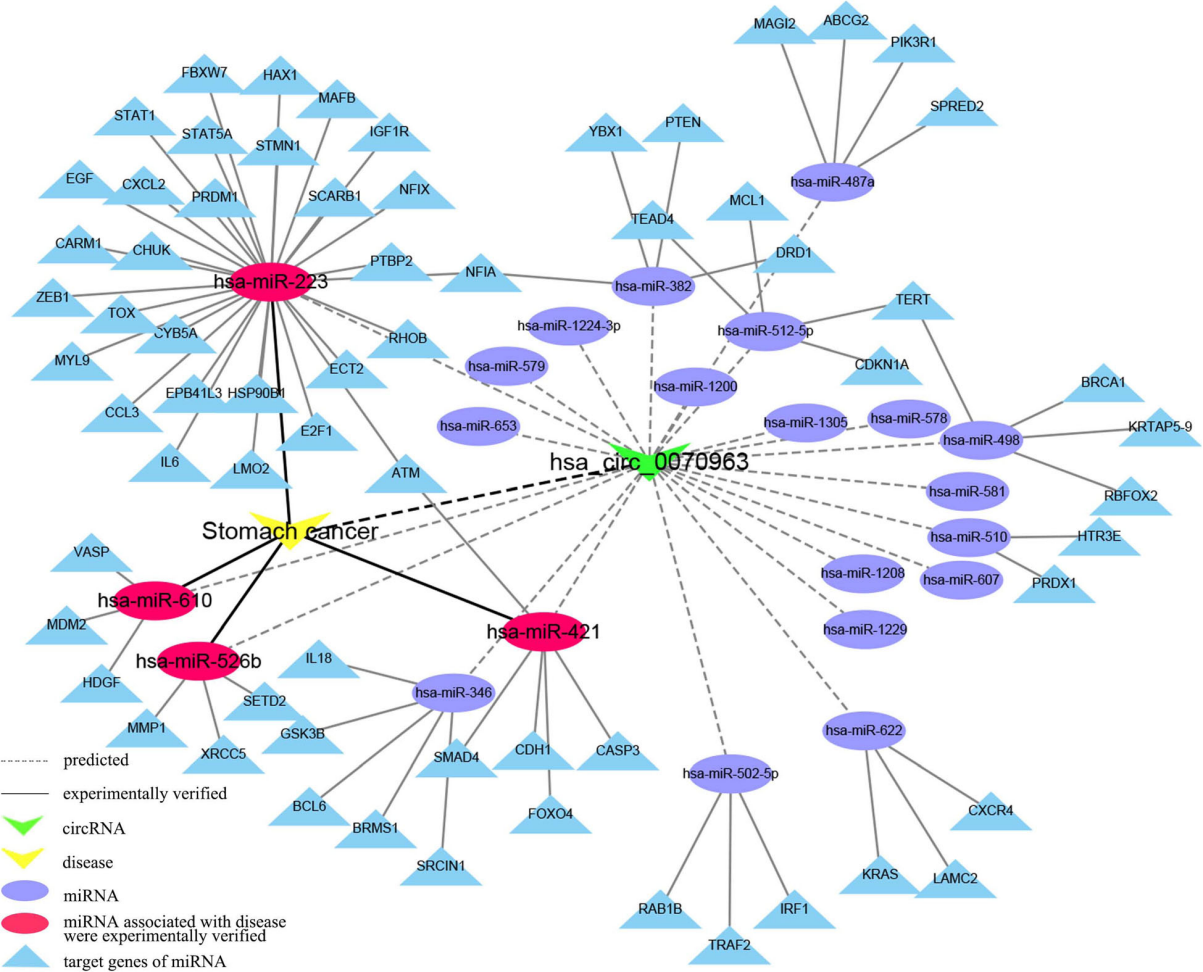
hsa_circ_0070963-miRNA-mRNA regulatory network in stomach cancer

We used the hsa_circ_0070963-stomach cancer association as an example for a detailed exposition. First, possible miRNA targets of hsa_circ_0070963 were predicted with the miRNA Target Sites tool of CircInteractome [40]. Their target genes with experimental verification were screened out from miRTarBase [41], and then, hsa_circ_0070963-miRNA-disease regulatory network was constructed using Cytoscape [42]. Finally, the corresponding experimentally verified miRNA-stomach cancer associations were obtained from HMDD [43] and added to the above network. As noted from the result (Fig. 6), hsa_circ_0070963 may be targeted by four miRNAs, including has-miR-223, has-miR-421, has-miR-610 and has-miR-526b. CircRNA can act as competing endogenous RNAs (ceRNAs) (also termed miRNA sponges) to buffer the target genes expression (i.e., mRNA) of miRNAs [36, 37], and miRNA has-miR-223 is linked the most number of targets. Thus, we hypothesize that hsa_circ_0070963 may function as a hsa-miR-223 sponge to interact with stomach carcinoma.

Other three predicted new associations (hsa_circ_0061893, hsa_circ_0071410, and hsa_circ_0054345 in stomach cancer) exhibit similar scenarios, which are presented in Additional file 1: Figures S9-S11.

## Conclusions

Increasing evidence demonstrates that circRNA plays an important role in the development of various diseases. Understanding the underlying mechanisms of circRNA in disease is becoming an urgent problem worldwide. To date, the number of experimentally validated circRNA-disease associations is small, and few computational methods for predicting circRNA-disease associations are available. In this paper, we proposed a method called SIMCCDA for predicting circRNA-disease associations based on known circRNA-disease associations. Integrating data regarding circRNA similarity and disease similarity, we employed IMC to construct the model. LOOCV was applied to assess the accuracy of the SIMCCDA. We then compared our method with KATZHCDA. Further case studies were also performed on breast cancer, stomach cancer and colorectal cancer. Based on the prediction results, SIMCCDA performs well in cross validations on the four datasets we used. Simultaneously, the compared results indicate that our method can identify more associations between circRNA and disease.

The prominent performances of SIMCCDA may have been facilitated by the following factors. First, SIMCCDA was constructed based on the integrated circRNA and disease similarities, which can make a full use of various similarity data to characterize potential circRNA-disease associations. Second, SIMCCDA transformed circRNA-disease associations into a recommendation system problem and applied the IMC algorithm of the recommendation system to predict potential circRNA-disease associations. A decisive advantage of IMC is that it can supplement the missing values in the circRNA-disease association matrix to improve the performance. Third, the datasets used in this study were derived from various validated databases. Observing the results obtained on the four datasets, we found that the prediction ability of our model was better than the previous method.

However, our model also has some limitations. First, although we introduce the sequence similarity of circRNA and the semantic similarity of disease, the calculation of Gaussian interaction profile kernel similarity relies heavily on known circRNA-disease associations, thus causing inevitable bias towards well-investigated circRNAs and diseases. Second, SIMCCDA could not be applied to unknown circRNA and diseases. In our future work, we will extend our method to solve these limitations.

## Supplementary information

**Additional file 1.** Supplementary file to this work (**Table S1-S2** and **Figures S1-S11**).

## Data Availability

The data pertaining to the present study has been included in table and/or figure form in the present manuscript. And all datasets and computational code underlying this study are available in an online archive https://github.com/bioinformaticsAHU/SIMCCDA.

## Abbreviations

AUC: Area under ROC curve
circRNA: circular RNA
DO: Disease Ontology
DOIDs: Disease ontology identities
IMC: Inductive matrix completion
lncRNA: long non coding RNA
LOOCV: Leave-one-out cross validation
miRNA: microRNA
PCA: Principal component analysis
PR: Precision-Recall
ROC: Receiver Operating Characteristics
SIMCCDA: Speedup Inductive Matrix Completion for CircRNA-Disease Associations prediction
SVD: Singular value decomposition
