# Supplementary material for: Prediction of circRNA-disease associations based on inductive matrix completion
Source: BMC Med Genomics. 2020 Apr 3;13(Suppl 5):42. doi: 10.1186/s12920-020-0679-0 (PMC7118830; doi:10.1186/s12920-020-0679-0)
Supplement: Supplementary file 1 — Additional file 1. Supplementary file to this work (Table S1-S2 and Figures S1-S11). [file 12920_2020_679_MOESM1_ESM.pdf]

Table S1. Details of four datasets (Dataset-4, Dataset-5, Dataset-6 and TotalCircRD-2**).**

Datasets	Number of circRNAs	Number of diseases	Number of associations	Matrix density
Dataset-4	310	40	331	0.026
Dataset-5	237	56	265	0.019
Dataset-6	459	68	516	0.016
TotalCircRD-2	683	89	789	0.012

Table S2. The number of associations validated by our model for the top 10, 30, 50 and 100 on eight datasets (Dataset-1, Dataset-2, Dataset-3, TotalCircRD-1, Dataset-4, Dataset-5, Dataset-6 and TotalCircRD-2).

	Top 10	Top 30	Top 50	Top 100
**Dataset-1**	7	26	46	64
**Dataset-2**	10	30	39	53
**Dataset-3**	10	30	50	84
**TotalCircRD-1**	10	30	46	84
**Dataset-4**	10	30	49	96
**Dataset-5**	10	30	50	79
**Dataset-6**	10	30	50	67
**TotalCircRD-2**	10	30	50	100

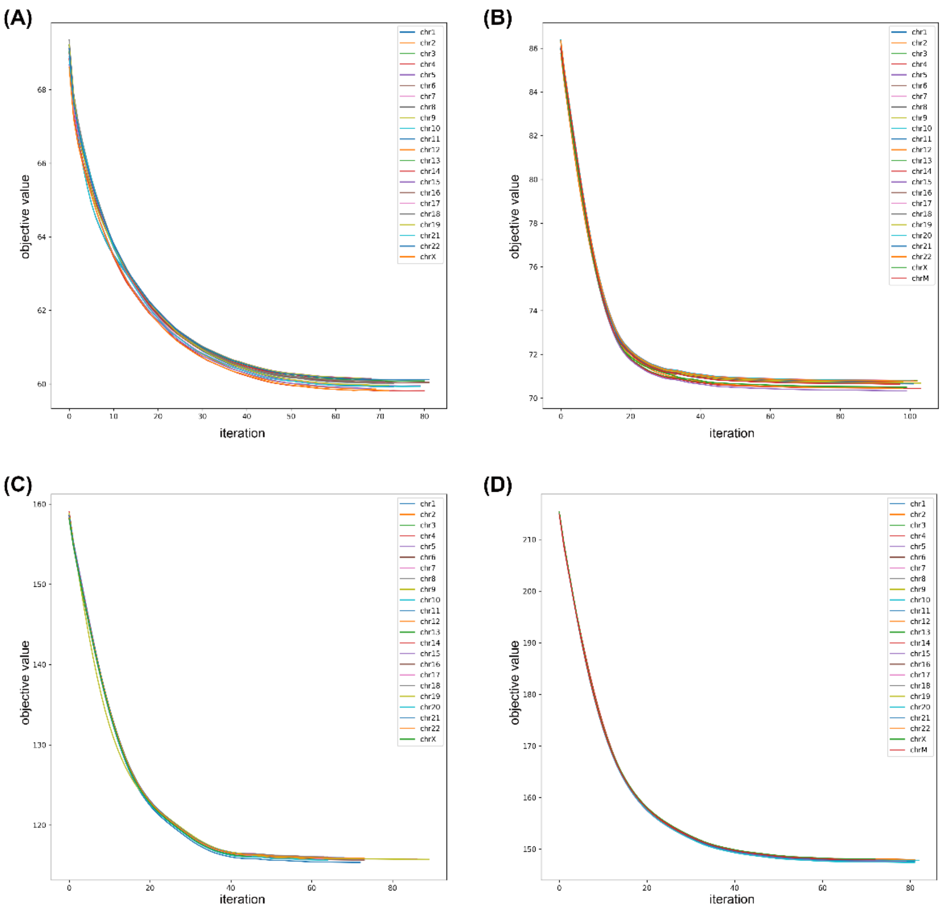

Figure S1. Objective function values versus iteration number in Dataset-1 (A), Dataset-2 (B), Dataset-3 (C) and TotalCircRD-1 (D).

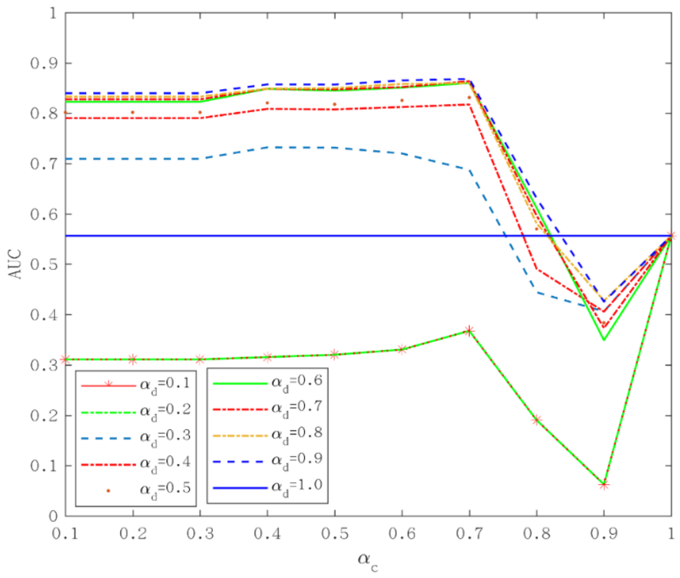

Figure S2. Adjust parameters α_c and α_d to determine the impact on Dataset-1.

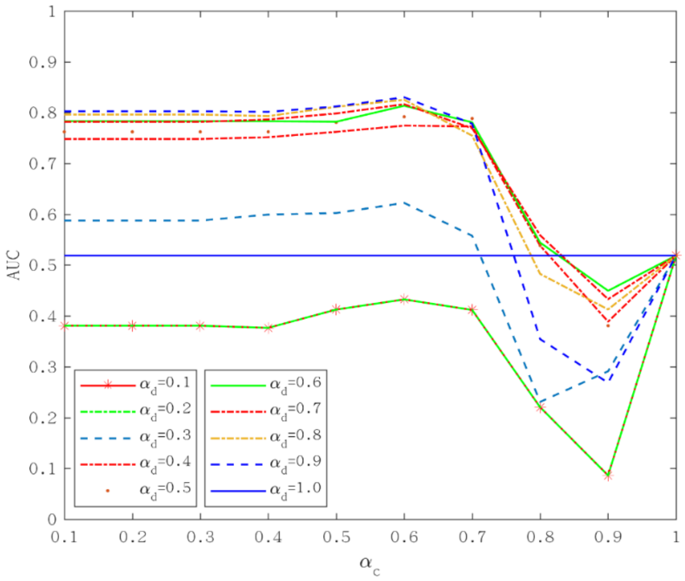

Figure S3. Adjust parameters α_c and α_d to determine the impact on Dataset-2.

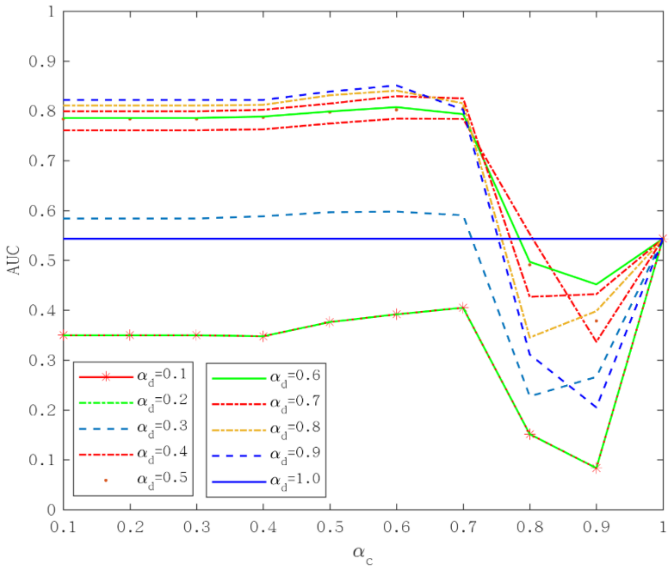

Figure S4. Adjust parameters α_c and α_d to determine the impact on Dataset-3.

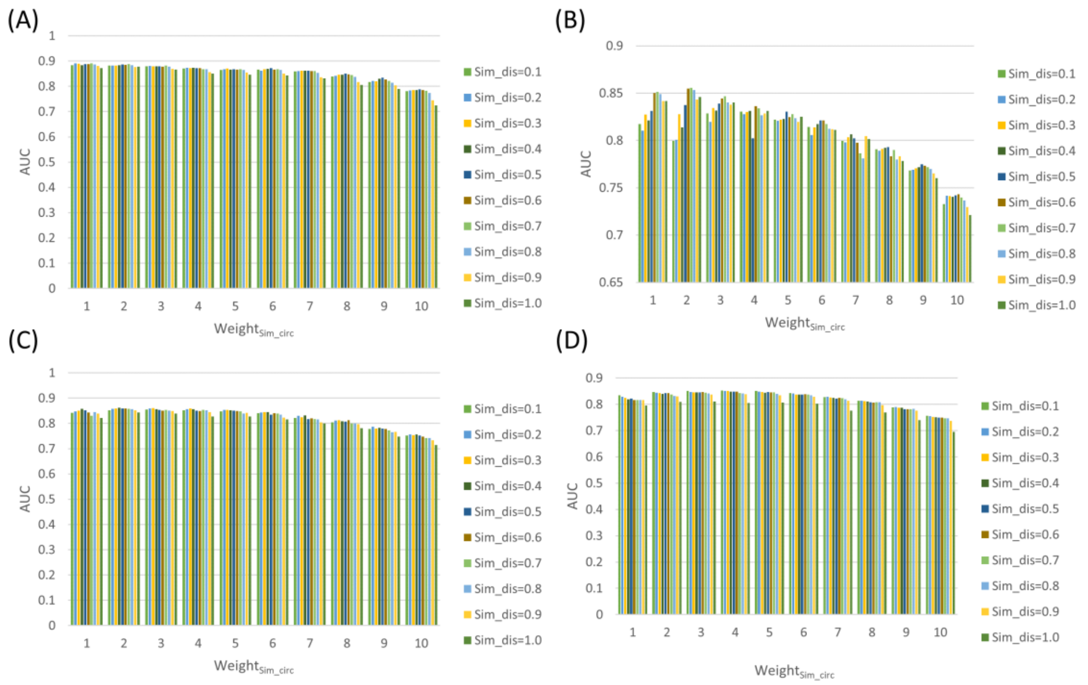

Figure S5. Different weight to similarity in four datasets (Dataset-1 (A), Dataset-2 (B), Dataset-3 (C), TotalCircRD-1 (D)). Weight$_{Sim_circ}$ represents the weight of $Sim_{lev}(circ_i, circ_j)$ which equals 1 minus the weight of $Gkl(circ_i, circ_j)$, Sim_dis represents the weight of $Sim_{Wang}(d_i, d_j)$ which equals 1 minus the weight of $Gkl(d_i, d_j)$.

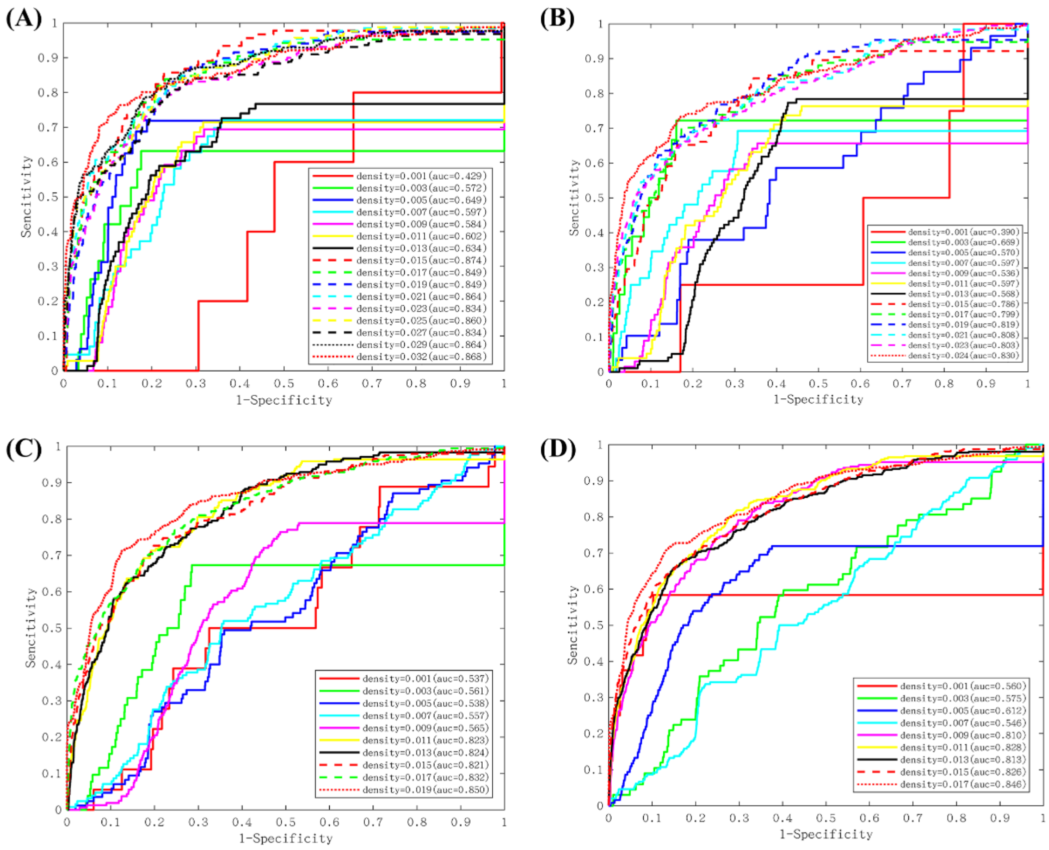

Figure S6. The effect of different sparsity on the results on the four datasets. (A) Dataset-1 dataset, (B) Dataset-2 dataset, (C) Dataset-3 dataset and (D) TotalCircRD-1 dataset.

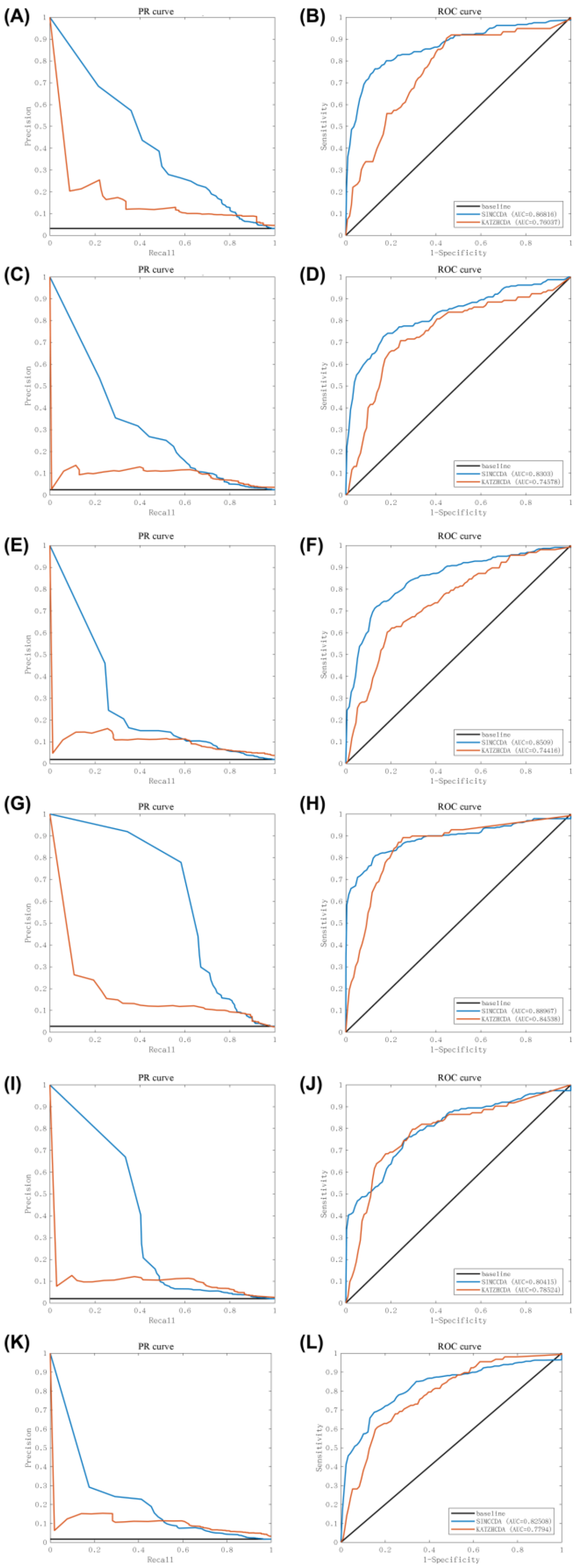

Figure S7. Comparison of SIMCCDA with KATZHCDA on the Dataset-1 (A, B), Dataset-2 (C, D), Dataset-3 (E, F), Dataset-4 (G, H), Dataset-5 (I, J) and Dataset-6 (K, L) using LOOCV-based PR curve and ROC curve.

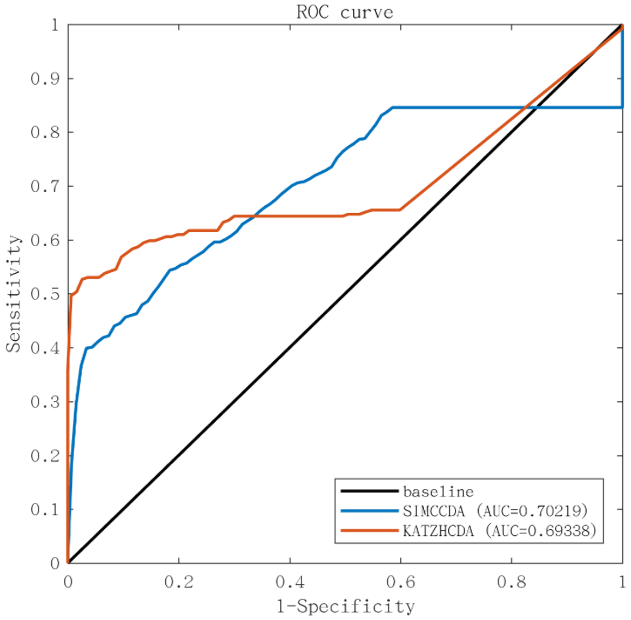

Figure S8. Comparison of SIMCCDA with KATZHCDA on the merged Dataset-2 and Dataset-3 (trained on Dataset-1).

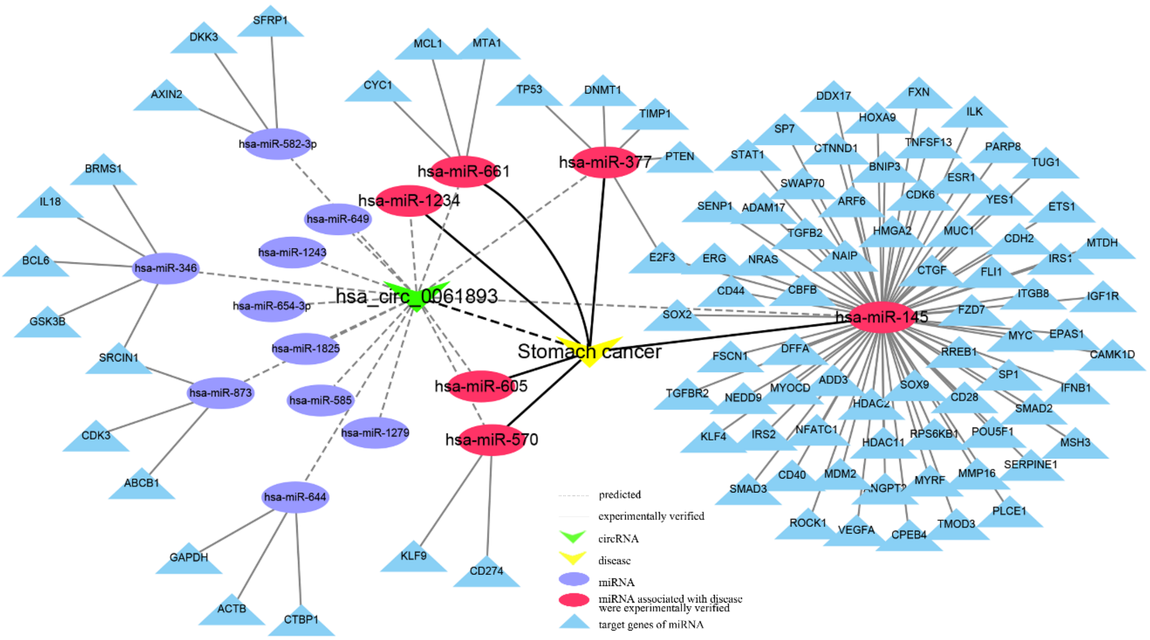

Figure S9. hsa_circ_0061893-miRNA-mRNA regulatory network in stomach cancer.

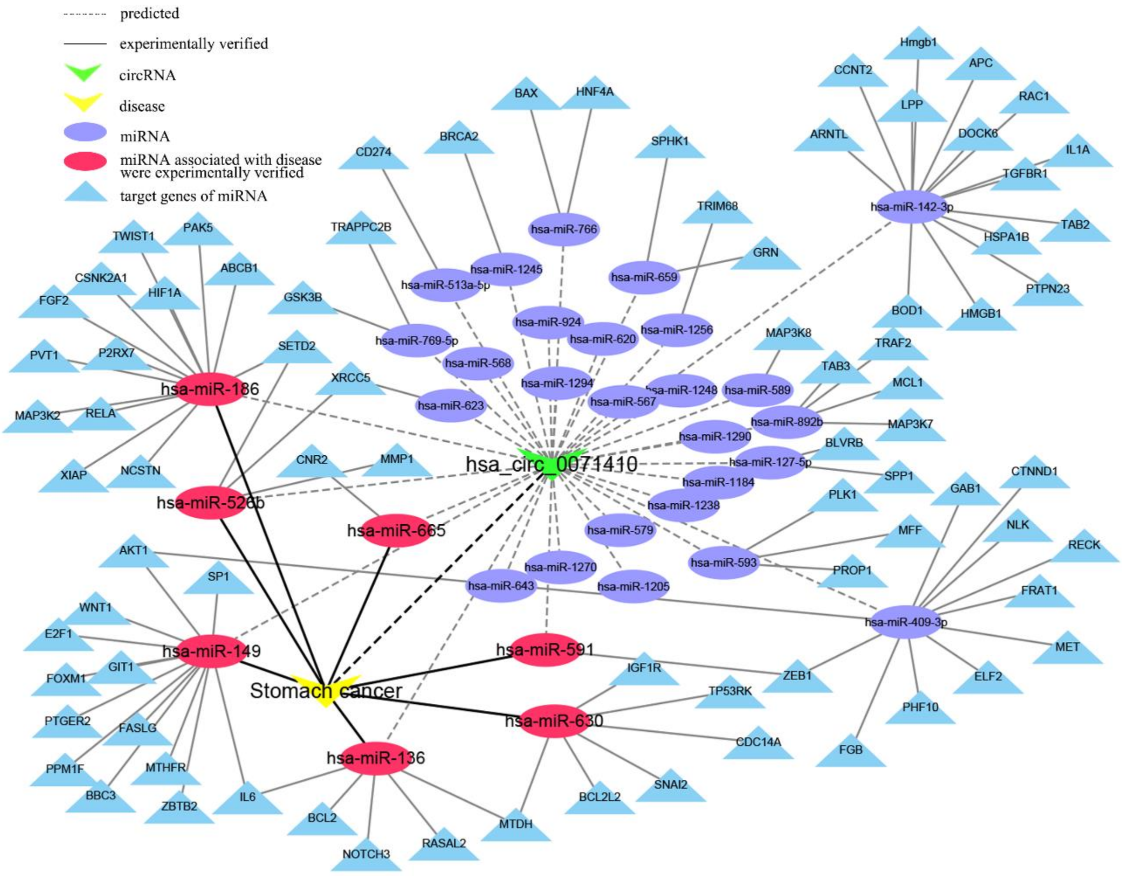

Figure S10. hsa_circ_0071410-miRNA-mRNA regulatory network in stomach cancer.s

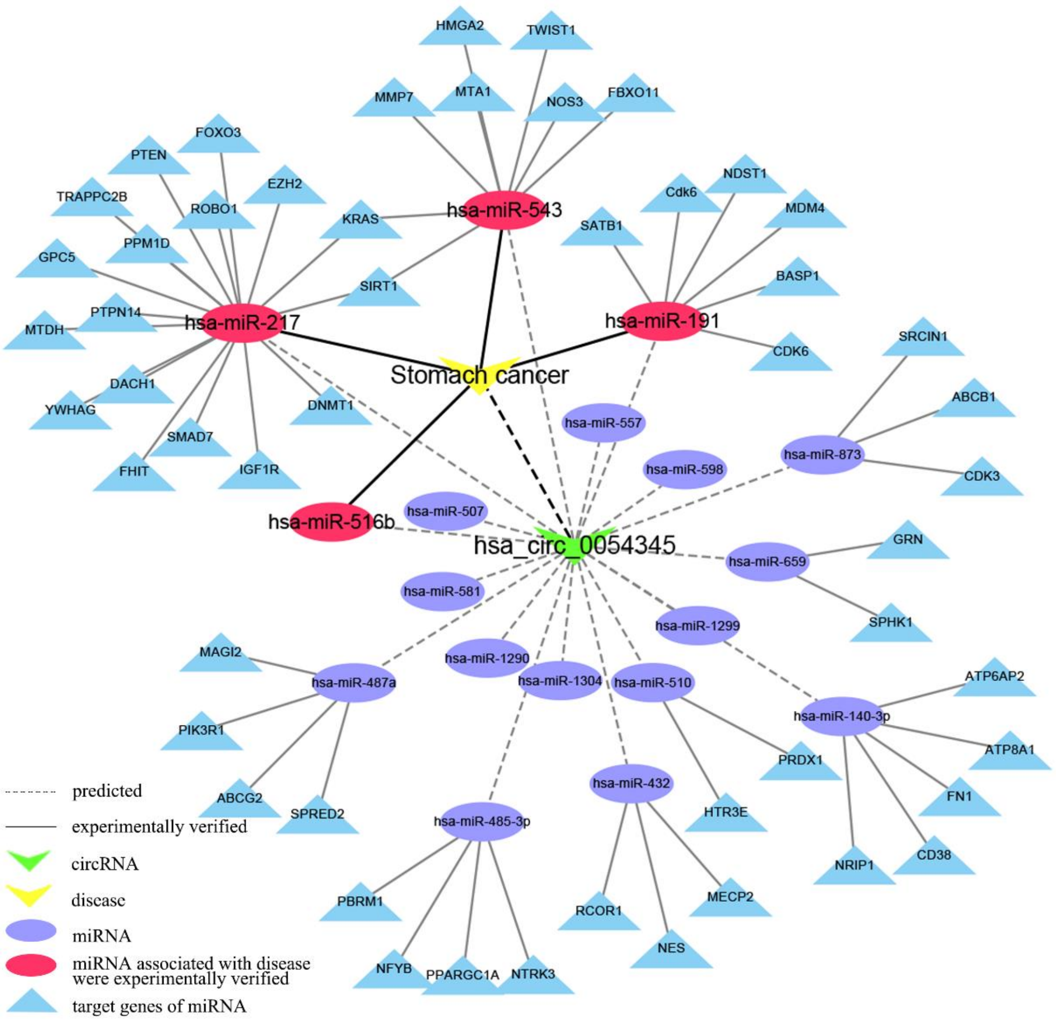

Figure S11. hsa_circ_0054345-miRNA-mRNA regulatory network in stomach cancer.
